# Sports injuries patterns in children and adolescents according to their sports participation level, age and maturation

**DOI:** 10.1186/s13102-022-00431-3

**Published:** 2022-03-09

**Authors:** Lara Costa e Silva, Júlia Teles, Isabel Fragoso

**Affiliations:** 1 Laboratory of Physiology and Biochemistry of Exercise, Faculty of Human Kinetics, University of Lisbon, Lisbon, Portugal; 2 CIPER, Faculty of Human Kinetics, University of Lisbon, Lisbon, Portugal; 3 Mathematics Unit, Faculty of Human Kinetics, University of Lisbon, Lisbon, Portugal

**Keywords:** Sports injuries, Children and adolescents, Bone age, Peak height velocity, Sports participation level

## Abstract

**Background:**

Growth can make young athletes more vulnerable to sports injuries. Increased knowledge about injury profile and its predictors is an important part of an overall risk management strategy but few studies have produced information.

**Methods:**

Information about injury profile and sports participation (SP) level was obtained by LESADO and RAPIL II questionnaires. They were distributed to 651 participants aged between 10 and 18 years attending four schools. Maturity measures were evaluated through maturity offset (MO) and Tanner-Whitehouse III method. Bivariate analysis was used to identify the set of candidate predictors for multinomial logistic regression analysis that was used to determine significant predictors of injury type and body area injury location.

**Results:**

Regarding injury type predictors recreative boys had more chances of having a sprain or a fracture than a strain. Also, recreative and scholar girls had more chances of having a sprain than a strain. As MO decreased, the chances of girls having a strain or a fracture when compared to sprains were higher. For body area location boys with 10–11 years were more likely to have upper limbs injuries than boys of other ages. This was also confirmed by MO. Spine and trunk injuries were more likely to occur in federate and no sports participation girls.

**Conclusions:**

Injury type and body area injury location differed significantly by SP level, age group and MO.

## Introduction

Musculoskeletal injuries are the most common injuries in youth sports [1]. Growth spurt, maturity-associated variation and lack of complex motors skills needed for certain sports are among the risk factors that may play an important role in the growing athlete [2, 3]. An epidemic of both acute and overuse injuries has been considered, as children make the transition for adolescence [4]. Enhanced environment for injury can occur and several studies reported structural and tissue changes that may contribute to this situation [1, 3–8]. Asynchronous development of bone and soft tissue take place due to the rapid expansion of bones while growing [9]. The soft tissues do not follow this rapid bone growth and elongate slowly and passively, thus becoming progressively tighter [4, 7, 8]. Although controversial for some authors, loss of flexibility may occur [3, 8] and tension develops across growth plates, apophyses, muscle–tendon units and joints. This increase in tensile forces can place these structures at risk of injury [7, 10]. Also an imbalance between strenght and flexibility can happen. The period in which trunk length and leg length have already increased, but muscles still have not reached their full size, lack of strength can become a potential cause of injury. This may lead to abnormal movement mechanics and to a motor performance decline during peak height velocity (PHV) [11]. Moreover children’s bones are weaker [7], because bone mineralization may lag behind linear growth, thus rendering the bone temporarily more porous [8]. Therefore, there is an increased risk for fractures throughout the bone and growth plate [5, 7, 12], confirmed through the association between PHV and peak fracture rate [3, 12, 13]. Likewise, biomechanical and clinical evidence suggests that growth cartilage is less resistant to repetitive microinjury [4, 5, 8, 9, 12]. During PHV, growth plate is less resilient to traction and compression forces because it´s predominantly composed by metabolically active chondrocytes, rather than by extracellular matrix [10]. Also, during adolescence, a decrease in coordination and balance may occur, which not only increases the risk of injury, but also influences sports performance [7]. All these events acting singly or together make the immature musculoskeletal system less able to cope with trauma situations and repetitive biomechanical stress [4, 8]. Another factor that also has to be issued is maturity-associated variation. Children of the same chronological age may vary considerably in biological maturity status which can make individual differences appear, creating unbalanced competition between early and late maturers contributing to serious injuries [3, 14]. Some studies already pointed to the fact that about 1/3 of all players of one age category are not within their normal maturity category [15].

As children and adolescents participate in sports in record numbers, targeting risk groups is important [3]. Also, increased knowledge about injury profile and its predictors, associated with specific physical activity (PA) exposures is an important part of an overall risk management strategy [16]. So, our aim was to determine injury type and body area injury location predictors in Portuguese youth, engaged in four different SP levels.

## Materials and methods

Ethics Committee of the Faculty of Human Kinetics approved the research protocol. The recommended principles set by the Helsinki Declaration for scientific research involving human beings were also followed, and before inclusion in the study all subjects’ guardians gave their written informed consent. STROBE cross sectional reporting guidelines were followed [17].

LESADO and Rapil II questionnaires were distributed to 651 participants in four schools, aged between 10 and 18 years involved in different levels of sports participation. LESADO is a self reported questionnaire that gathers information about injury profile. It comes from an extensive literature review on the topic and was adapted and based on epidemiological questionnaires used in Portuguese sports samples [18–22]. As our subjects were children and adolescents, the time to fill out the questionnaires was supervised by the investigator who followed and clarified all doubts, preventing the possibility of bias and interpretation difficulties associated with literacy skills [23, 24]. A clear definition of injury and selected variables was provided, based in current epidemiological research [25, 26], and can be consulted in previous studies [18, 19, 27]. Time frame used was six months, as recommended in retrospective studies [28, 29]. The Biosocial Questionnaire RAPIL II is a parent´s self-reported instrument and it was used to measure biosocial variables. It´s been used in Portugal in large epidemiological studies [30–32], and provides information about the daily PA habits of the subject. These data allowed to create four groups of SP. The no sports participation group, with no time spent in PA per week (except mandatory physical education classes), the recreative sports group with at least 90 min of PA per week being at least 60% of this volume of recreational sports activity; the school sports group with at least 90 min of PA per week being at least 60% of this volume of school sports activity and the federated sports group with at least 120 min of federated activity. Federated sports athletes are also defined as those who have official recognition for their sport by a sanctioned sports association. These athletes usually have medical approval to participate and formal training/coaching. School sports are understood as the set of recreational-sports and training practices with a sporting objective developed as a complement to the curriculum and occupation of free time, integrated into the school's activity plan and coordinated within the scope of the education system.

On the other hand, recreational sports involve one or more participants and provides fun and entertainment for participants in a non-formal/structured practice setting. The competitive value is minimized and the rules can be changed depending on the objective of the game/sport.

Maturity measures consisted in calculating bone age and maturity offset. Bone age was obtained according to the Tanner-Whitehouse III (TW3) method [33]. Radiographs of left hand and wrist were taken in a single session, and the maturity ratings of thirteen bones were performed by one trained examiner, without knowledge of the chronological age of the subjects.

Maturity offset assessed time before or after PHV according to Mirwald [34]. Maturity offset minus chronological age provides an estimate of the age of PHV. It can be used to group the individuals for years before or after PHV. We used a specific equation for each sex (SEE equation is 0.592 for boys and 0.569 for girls), based on the Canadian and Belgian samples [34]. Applicability of the method appears to be useful during the period of growth acceleration, between 12–15 years [35]. Chronological age group was defined with the whole year as the midpoint of the range (e.g., 12 years include participants with 11.50–12.49 years of decimal age).

The statistical analysis was conducted using SPSS 22.0 software (SPSS Inc., Chicago, IL, USA) and a significance level of 5% was considered. The dependent variables were injury type (0 = strain, 1 = sprain 2 = fracture) and body area injury location (0 = lower limbs, 1 = upper limbs, 2 = spine and trunk). Despite some issues that prevent the use of multinomial regression models in case of body area injury location, this technique was initially considered to identify the significant predictors for each sex and for each dependent variable. The evaluated predictors were SP level (0 = no sports participation, 1 = recreative, 2 = scholar, 3 = federate), age group (0 = 10–11, 1 = 12–13, 2 = 14–15, 3 =  ≥ 16 years), bone age (years) and maturity offset (years). First bivariate analyses of predictors were conducted: for each dependent variable chi-square tests of independence and Kruskal–Wallis tests were used with categorical and quantitative predictors, respectively. The set of candidate predictors for multinomial regression consisted of all the variables that presented *p* < 0.25 in the bivariate analysis [36], and the backward stepwise method using the likelihood ratio statistic was applied in the model variable selection.

## Results

The sample included 651 adolescents, aged between 10 and 18 years (Mean = 13.7; Standard Deviation = 1.8 years), being 343 boys (52.7%) and 308 girls (47.3%). A total of 247 subjects reported a sports injury during the previous 6 months (37.9%; 95% CI 34.2–41.7%). Considering the analysis by sex, 143 of 343 boys reported an injury (41.6%) and 104 of 308 girls reported an injury (33.8%). Results of the injury profile are presented in Table 1.

**Table 1:** - Prevalence and injury profile

	Frequency	Percentage
Injury prevalence	247	37.9
**Body area location**		
Lower limbs	107	53.8
Upper limbs	58	29.0
Column and Torso	23	11.5
**Injury type**		
Strains	67	33.7
Sprains	54	27.1
Fractures	46	23.1
**Ocurrences**		
Practice	174	74
Competition	61	26
**Causes**		
Direct trauma	123	51.9
Indirect trauma	70	29.5
Oversuse	30	12.7
**Classification**		
1st injury	123	51.9
Relapse	59	25
Chronic	38	15.9
**Consequences**		
Total recovery	143	60.9
Conditioned activity, symptoms or treatments	92	39.1
**Severity**		
< 1 week	135	54.6
≥ 1 week	112	45.4

### Boys—predictors of injury type

Significant associations were found only between injury type and SP level (X^2^(4) = 12.763, *p* = 0.011). Backward stepwise methods lead to a multinomial logistic regression model (X^2^(4) = 15.165, *p* = 0.004). The odds of a recreative boy having a sprain rather than strain were 8.84 times more than for a federate boy and the odds of a recreative boy having a fracture rather than a strain were 7.27 times more than for a federate boy. Results can be seen in Table 2.

**Table 2:** Multinomial logistic regression models adjusted for the dependent variable injury type for each sex

Dependent variable	Predictor	*B* (Std error)	*p*	odds ratio	95% CI odds ratio
Type of injury^1^		Boys^3^			
Sprain	Intercept	− 0.793 (0.276)	.004		
	SP level (0)	0.100 (0.672)	.882	1.105	(0.296, 4.125)
	SP level (1)	2.180 (0.838)	.009	8.842	(1.713, 45.651)
Fracture	Intercept	− 0.480 (0.250)	.055		
	SP level (0)	− 1.600 (1.090)	.142	0.202	(0.024, 1.709)
	SP level (1)	1.984 (0.821)	.016	7.269	(1.455, 36.306)
Type of injury^2^		Girls^4^			
Strain	Intercept	2.272 (0.810)	.005		
	Maturity offset	− 0.538 (0.224)	.016	0.584	(0.376, 0.906)
	SP level(0)	− 1.249 (0.756)	.098	0.287	(0.065, 1.262)
	SP level(1)	− 2.012 (0.824)	.015	0.134	(0.027, 0.673)
	SP level(2)	− 3.029 (1.239)	.015	0.048	(0.004, 0.549)
Fracture	Intercept	2.050 (0.895)	.022		
	Maturity offset	− 0.842 (0.253)	< .001	0.431	(0.262, 0.707)
	SP level(o)	− 1.869 (0.974)	.055	0.154	(0.023, 1.041)
	SP level(1)	− 1.541 (0.932)	.098	0.214	(0.034, 1.330)
	SP level(2)	− 0.572 (0.945)	.545	0.564	(0.089, 3.596)

^1^The reference category is strain

^2^The reference category is sprain

^3^Model *X*^2^(4) = 15.165, *p* = .004; Cox & Snell *R*^2^ = .120; Nagelkerke *R*^2^ = .135; McFadden *R*^2^ = .059

^4^Model *X*^2^(8) = 28.770, *p* < .001; Cox & Snell *R*^2^ = .290; Nagelkerke *R*^2^ = .328; McFadden *R*^2^ = .158

### Girls—predictors of injury type

**Fig. 1:**
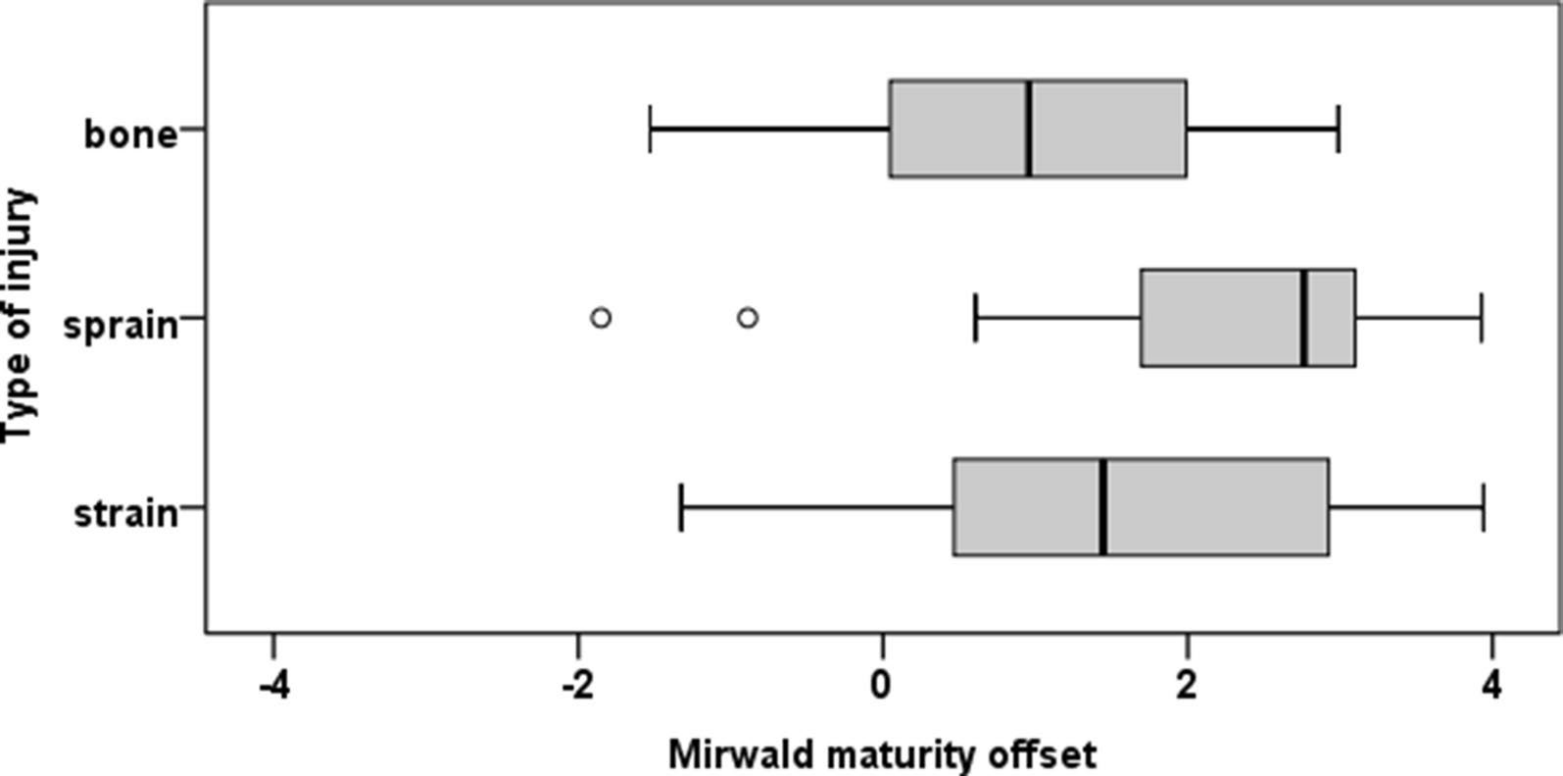
Boxplots of maturity offset for girls by type of injury

Regarding girls, Kruskal–Wallis tests showed that there were significant differences in bone age (X^2^(2) = 9.616, p = 0.008) and maturity offset (X^2^(2) = 12.892, p = 0.002) among injury type. Although SP level (X^2^(6) = 12.117, *p* = 0.059) was only marginally significant predictor in the bivariate analysis, it was considered as candidate predictor for the multinomial logistic regression since together with other predictors could be significant, as it happened. The multinomial logistic regression model achieved two predictors, SP level (X^2^(6) = 16.474, p = 0.011) and maturity offset (X^2^(2) = 15.115, p < 0.001). The odds of a recreative girl having a sprain rather than a strain were 7.46 (1/0.134) times more than a federate girl and the odds of a scholar girl having a sprain rather than a strain were 20.8 (1/0.048) times more than a federate girl. Relatively to maturity offset, the odds ratio revealed that as maturity offset decreased by a unit, the change in the odds of having a strain rather than a sprain were 1.71 (1/0.584); and of having a fracture rather than a sprain were 2.32 (1/0.431). Results are presented in Table 2 and Fig. 1.

### Boys—predictors of body area injury location

**Fig. 2:**
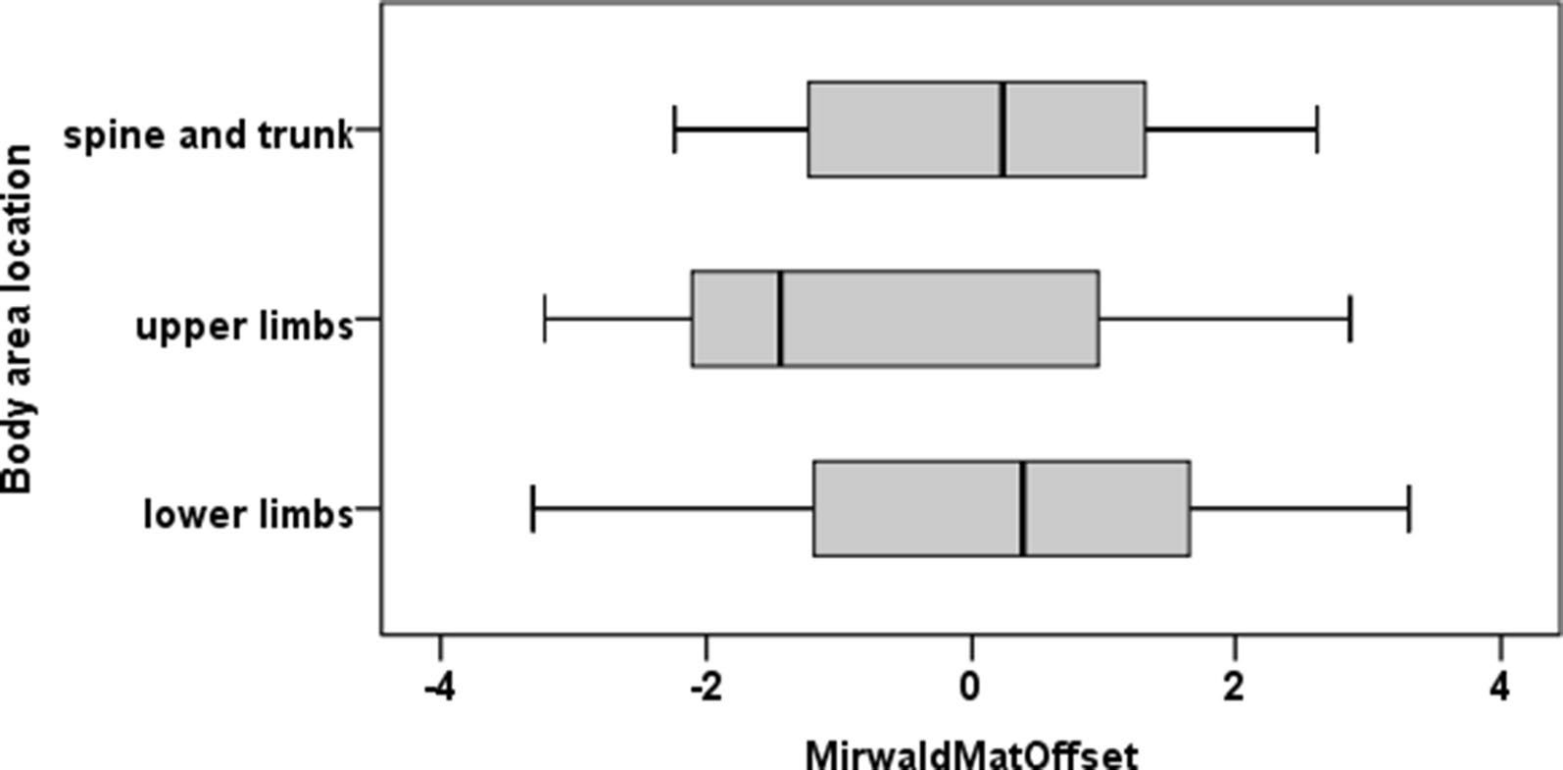
Boxplots of maturity offset for boys by body area injury location

A significant association was found between body area injury location and age group (X^2^(6) = 13.587, p = 0.033). Boys with 10–11 years were more likely to have upper limbs injuries than boys of the other age groups and less likely to have lower limbs injuries than boys of age groups 14–15 and ≥ 16. Kruskal–Wallis tests also revealed that significant differences emerged in maturity offset (X^2^(2) = 6.014, p = 0.049). Post hoc tests showed that the differences in maturity offset were between upper limbs and lower limbs (p = 0.045). See Fig. 2.

### Girls—predictors of body area injury location

**Fig. 3:**
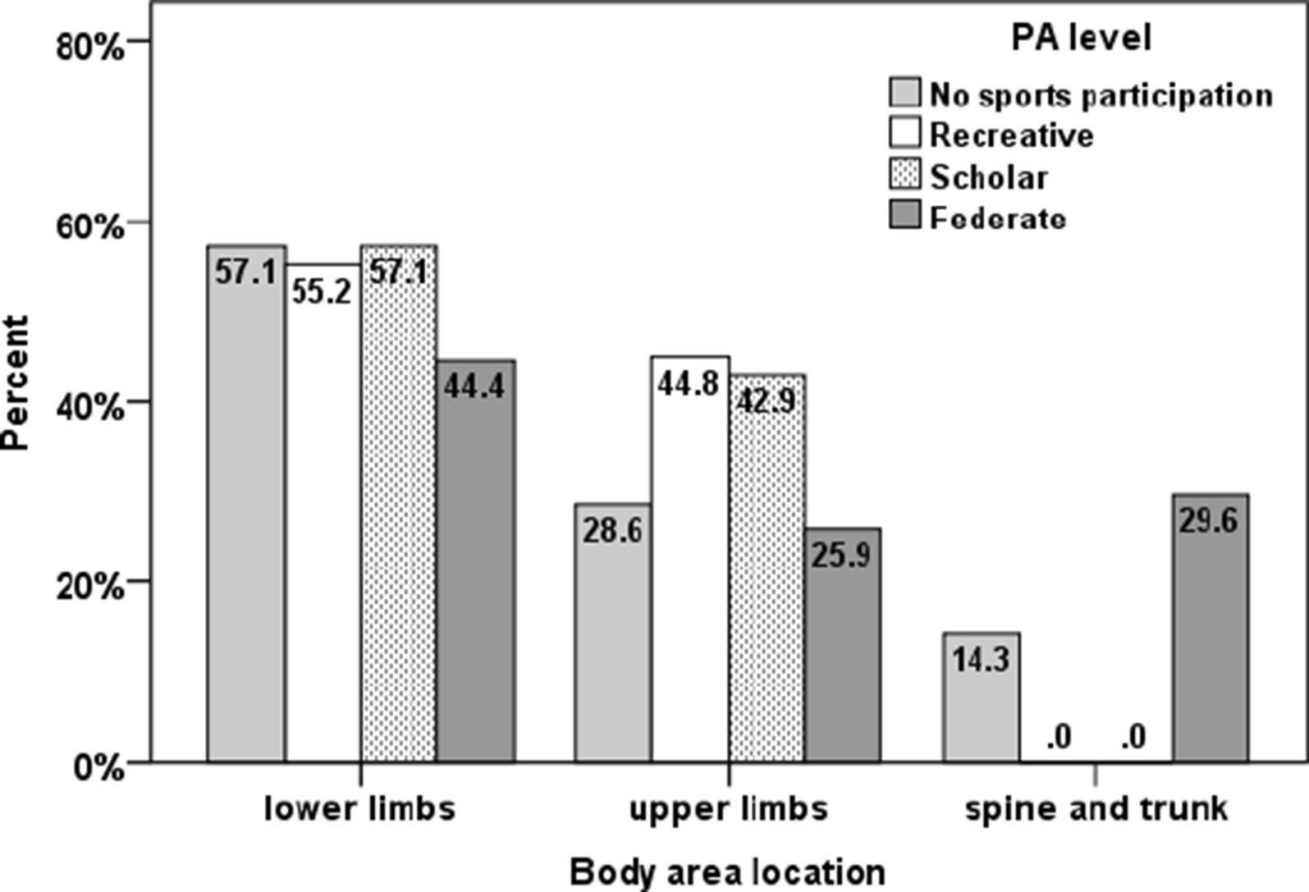
Girls percentage of injuries by body area injury location for each SP level

A significant association was detected between body area injury location and SP level (X^2^(6) = 14.587, p = 0.022). Federate girls were more likely to have spine and trunk injuries than scholar and recreative girls, and girls with no sport participation were more likely to have spine and trunk injuries than recreative girls. See Fig. 3.

The reduced number of spine and trunk injuries for both boys and girls prevented the use of multinomial logistic regression in case of body area injury location.

## Discussion

Injuries in school age children from different PA backgrounds have a specific identity [18, 19, 37], being age, SP level and maturation important predictors of body area injury location and injury types. Each sport group presented a specific injury profile and PHV proved to be an important milestone for the evaluation of the injury pattern in adolescents of both sexes. Due to the variation observed in growth and maturation between adolescents, chronological age turns out to be a less informative indicator for injury risk. Inter-individual biological maturation variability, corresponds to inter-individual readiness for sport acquisitions and specific vulnerability to certain injuries.

### Sports participation level

The distribution through the different levels of sports participation seems to be one of the key variables in regard to injury type. Scholar girls were more likely to have sprains rather than strains 20.8 times. Like it was proven by several studies, sprains are one of the most common injuries sustained by young athletes [2, 37] and highly related with traumatic mechanisms [38], due to joint stiffness and abnormal movement mechanics during growth [11]. In addition, concerns about school sports have been raised due to the poor quality of the playing fields, inappropriate protective equipment and inefficient supervision [6], which may also explain the traumatic nature of injuries found in this group. Boys and girls of the recreative group were 8.84 and 7.46 times respectively more likely to have sprains than strains, and boys had 7.27 times more chances of having a fracture rather than strains. The concerns raised in regard to scholar sports about environmental, equipment and supervision issues are also present in recreative sports. Also, recreative sports can be practiced in a variety of settings, which can add complexity to injury patterns. As fractures in boys are concerned, younger males tend to sustain during sports practice, more accidental injuries, especially fractures, than girls, older children and adults [39]. The high incidence of fractures in childhood result from a transient deficit in bone mass related to longitudinal growth [13].

Federated girls and boys reported more strains, rather than sprains or fractures. Federated athletes suffer a great amount of soft tissue injuries being the majority of them classified as overuse. Recent studies are beginning to emphasize and describe overuse injuries as the most significant mechanism of injury in organized sports. The increasingly highly competitive nature of youth sports, increased periods of extensive training, repetitive movements, sport specialization and participation in large numbers of competitive events [19, 40, 41] have made overuse injuries a growing reality. In addition, structured sports training and competition do not always allow adequate rest periods for a developing child [10]. Subjects who have not developed some skills like strength, endurance, and motor control may be at increased injury risk as they begin or get more involved in a specific sport [12]. Also in organized competitions, the child may feel an expectation to continue and therefore be more likely to push through pain or soreness.

Girls in the no sports participation and federated groups presented more chances of having a spine or trunk injury (X^2^(6) = 14.587, p = 0.022). Low levels of PA and sedentary lifestyle can be considered a risk factor. Physical inactivity can result in decreased strength, bone mineral content, flexibility and coordination, increased body fat mass, and these factors can contribute to the appearance of symptoms, especially in girls [42–45]. On the other hand, it is also common scientific studies report young athletes as a risk group for spine dysfunction [46, 47]. Low back pain in athletes is usually directly related to sports practice. The protective effect of sport participation disappears and a detrimental effect manifests itself as a result of the high volumes and intensities of training. Functional or repetitive overload and/or charges early introduced, not adapted to the growth and physiological characteristics of the athlete are usually the main causes for low back injury [48, 49].

### Maturity offset

Considering MO, strains and fractures were more likely to occur in girls near the PHV. It´s consensual that around the PHV period, adolescents are vulnerable to injuries [14, 18, 50]. Physiological loading is beneficial for the bones, but excessive load may produce serious injuries, like strains [50].

An increase in traumatic injuries takes place mainly during the time of PHV, while the increase in overuse injuries persists in the year after PHV. A period in which trunk and leg length have already increased, but muscles still have to grow, to reach their full size and strength, an imbalance between strength and flexibility can occur. This imbalance, during PHV interval, may lead to abnormal movement mechanics and a decline in performance on motor tasks during the interval of PHV. Possibly, this temporarily decline in essential motor performance during the years of maximal growth contributes to an increase in traumatic injuries [11]. Additionally the decrease in bone mineral density before PHV correlates with acute fracture episodes [10–13]. Fractures during childhood and adolescence are more frequent in girls with later menarche rather than earlier menarche. These factors, reported as responsible for an increase in traumatic injuries (joint stiffness, decreased bone density, abnormal movement mechanics) disappear 1 year after PHV, in contrast to factors that contribute to overuse injuries. As overuse injuries are concerned, authors have explained its causes from a biomechanical perspective. First, changes in bones limb mass typically occur before visible changes in muscle tissue. If muscles, tendons and apophyses adapt slowly, and activities are performed repetitively, those tissues are not immediately able to deal with the increased stress and overuse injuries may occur, leaving a period of increased susceptibility after PHV [11]. Moreover, it should be noted that girls present higher overuse injury rates than boys [41]. Relatively to body area injury location, only boys presented significant results. Boys’ upper limbs injuries were more likely to occur before PHV, and lower limbs injuries after PHV (p = 0.045). These results reflect the relation between type of injury, growth velocity during adolescence, and body area injury location, where traumatic upper limb bony injuries can occur more often in children/adolescents before PHV, and soft tissue lower limb injuries in adolescents after PHV. It is known that significantly larger proportion of injuries sustained by older children are on soft tissues when compared with younger athletes. Younger athletes are more likely to have bone fractures, normally located in the upper limbs [26] and are treated for a greater amount of traumatic injuries [5]. During puberty, the asynchrony between the stature growth acceleration and bone mineral content is also seen in the distal radius with a transient cortical deficit with an increased porosity that may well contribute to the adolescent increased incidence in forearm fractures [13]. On the other hand, increased stress on the muscle–tendon-bone junctions, ligaments, and growth cartilage occurs as the changes in the length, mass, and moment of inertia of the extremities take place with growth. Although tissue and structural dynamic equilibrium begins to be reached after PHV some degree of fragility still persists. The increase in strength needed to accommodate these changes may not occur in a uniform pattern and may enable the child or teenager to continue to generate the same limb speed as before the growth spurt. These complex factors and combinations of growth, strength, load, sport training and competition create situations conducive to the development of overuse injuries, especially in lower limbs [12].

### Age group

Group age results reflect the maturation results. Boys with 10–11 years were more likely to have upper limbs injuries than boys of the other age groups and less likely to have lower limbs injuries than boys of age groups 14–15 and ≥ 16 (X2(6) = 13.587, p = 0.033). Some authors are starting to recognize that the effect of age on injury risk may be trivial at these ages [14, 18].

### Study limitations

One of the limitations of study studies lies on the retrospective methodology used for information collection. Relying on the participants’ correct memory of events can introduce recall bias, potentially leading to incorrect conclusions. Minimisation of recall bias is a prerequisite when the collection on self-reported data cannot be avoided. Providing a clear definition of injury can help to improve the memory of participants through the provision of specific prompts [25, 26]. Limiting the length of time over which participants are asked to recall injuries can also help to reduce the impact of the recall bias [29]. The samples’ group age also brought some limitations. Surveys have been shown to be useful for collecting children´s injury and sport participation data [24]. Children are able to give a detailed account of the circumstances of the injury event [51]. Nevertheless difficulties can be encountered when using survey measures with children [24]. One is their capacity to recall information and second is their low literacy skills. These technical problems can be prevented through a tight following when the survey is being completed [24].

## Conclusion

Some injury risk factors are unique to the growing athlete. Increased knowledge about injury characteristics associated with specific PA exposures and maturation variables are an important part of an overall risk management strategy. A specific injury profile was presented at all levels of sports participation. PHV was a significant predictor of injury patterns in adolescents of both sexes. Chronological age may not be a good indicator of injury risk and maturation assessment can be a more complete measure to estimate injury risk. Evaluation of biological maturation should be strongly encouraged.

## Data Availability

The datasets used and/or analysed during the current study are available from the corresponding author on reasonable request. They can be presented as supplementary information files.

## Abbreviations

SP: Sports participation
MO: Maturity offset
PHV: Peak height velocity
PA: Physical activity
SEE: Standard error of estimate
